# Clinical and Molecular Characteristics of 60 Patients With Human Immunodeficiency Virus-Negative Castleman Disease

**DOI:** 10.3389/fimmu.2022.899073

**Published:** 2022-05-17

**Authors:** Siyu Qian, Mengjie Ding, Huting Hou, Zeyuan Wang, Jieming Zhang, Yue Zhang, Meng Dong, Linan Zhu, Guannan Wang, Wencai Li, Xudong Zhang

**Affiliations:** ^1^ Department of Oncology, The First Affiliated Hospital, Zhengzhou University, Zhengzhou, China; ^2^ Department of Pathology, The First Affiliated Hospital of Zhengzhou University, Zhengzhou, China

**Keywords:** Castleman disease, classifications, prognosis, mTOR pathway, thrombocytopenia

## Abstract

Castleman disease (CD) is a rare lymphoproliferative disorder. The mechanistic target of rapamycin (mTOR) pathway is a key regulator of various cellular functions, which may be related with the potential mechanisms of CD occurrence. We retrospectively collected the clinical information of 60 CD patients diagnosed in the First Affiliated Hospital of Zhengzhou University. And FFPE biopsy specimens were collected from 31 patients (12 unicentric CD patients and 19 multicentric CD patients) to detect the mTOR pathway protein expression. We are the first to demonstrate that thrombocytopenia and hypoalbuminemia are independent poor prognostic factors for CD. Moreover, mTOR activation was higher in CD compared to reactive lymphoid hyperplasia (used as a control group). This study offers some elucidation for the management and treatment of CD patients.

## 1 Introduction

**Figure 1:**
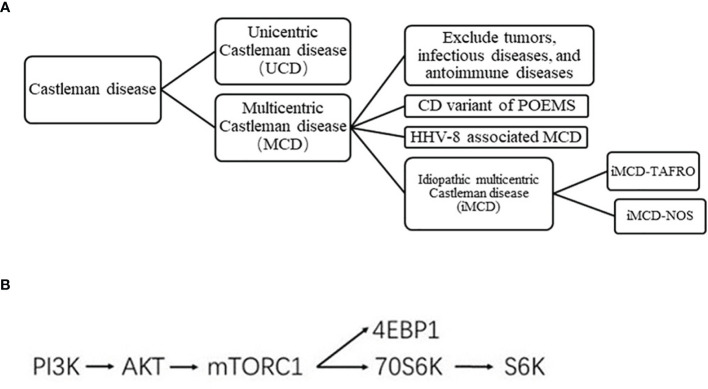
**(A)** Clinical classification of CD. **(B)** Simplified mTOR pathway.

Castleman disease (CD), also known as angiofollicular lymph node hyperplasia or giant lymph node hyperplasia, is a rare lymphoproliferative disorder first described by Benjamin Castleman in 1956 (1). According to the distribution of enlarged lymph nodes, it was clinically divided into unicentric CD (UCD) and multicentric CD (MCD). According to human herpes virus-8 (HHV-8) status, MCD was further classified as HHV-8 positive and HHV-8 negative; the latter is referred to as idiopathic MCD (iMCD) (2) (**Figure 1A**). TAFRO syndrome is a newly recognized variant of iMCD that includes a constellation of syndromes: thrombocytopenia, anasarca, fever, reticulin fibrosis, and organomegaly (3).

The clinical and mechanisms of CD remain poorly understood. UCD may involve a process of clonal tumorigenesis (4). The preferred therapy for UCD is complete surgical resection. Complete surgical excision usually eliminates all systemic symptomatology and laboratory abnormalities, if present (5). Interleukin 6 (IL-6) is a multifunctional cytokine *in vivo* that can stimulate bone marrow hematopoiesis and plasma cell differentiation, inducing the systemic symptoms of CD (6). HHV-8 encodes a viral IL-6 homolog with a similar structure and function to human IL-6, which leads to the occurrence of HHV-8-associated MCD (7, 8). As for HHV-8 associated MCD, treatment regimen based on rituximab can be used. Asymptomatic MCD patients can be followed up with only observation. There is no standard treatment for iMCD patients. Although iMCD patients tested negative for HHV-8, the level of IL-6 remained high. The role of excessive IL-6 signaling in the pathogenesis of iMCD has led to the development of novel iMCD therapies targeting IL-6. However, 50% to 66% of patients with iMCD remain refractory to therapy with an IL-6 blocker. The mechanistic target of rapamycin (mTOR) pathway is a key regulator of various cellular functions, such as protein synthesis and cell growth, division, and survival, and is a critical event in the development of different hematological malignancies (9). The mTOR inhibitor has been recommended as a second-line treatment for iMCD patients. At present, there is a lack of large number of clinical samples to explore the exact expression of mTOR pathway protein in CD patients. The present study evaluated the clinical characteristics of HIV-negative CD patients and determined the mTOR pathway activity using an immunohistochemical (IHC) approach.

## 2 Materials and Methods

### 2.1 Patients

From October 2012 to October 2021, 60 patients with CD confirmed through clinical, laboratory and pathological examination, with adequate follow-up data, from the First Affiliated Hospital of Zhengzhou University were recruited in this study. The patients’ data were obtained from the medical records and *via* telephone interview, including general information, clinical complaints and symptoms, results of pathological and IHC examinations of biopsy specimens, clinical laboratory tests, imaging performed, and follow-up information. None of the patients developed HIV infection. Follow-up was performed until December 10, 2020. Formalin-fixed paraffin-embedded biopsy specimens from 31 patients with CD (12 with UCD and 19 with MCD) and 10 patients with reactive lymphoid hyperplasia were collected for IHC analysis.

### 2.2 IHC

All tissues were embedded in paraffin, cut into 5-µm slices, and used for IHC analysis. After heating at 40°C for deparaffinization, the slices were deparaffinized three times (20 min each time) and rehydrated with absolute ethanol for 10 min, 95% ethanol for 5 min, and 80% ethanol for 5 min. The slides were incubated overnight with the primary antibodies against p70S6k, p4EBP1, pS6, S6, and pAKT. Immunostaining was performed by two independent pathologists.

### 2.3 Grading Criteria

At least 200 positive cells per field of vision were counted. The positive cells appeared yellow to dark brown in color. Based on the shade of positive staining, the sections were graded as follows: 0 points, no color; 1 point, light yellow; 2 points, tawny; and 3 points, dark brown. The amount of staining was graded as follows: 0 point, 0%–10% positive cells; 1 point, 11%–25% positive cells; 2 points, 25%–50% positive cells; and 3 points, >50% positive cells. The scores from the two evaluations were then multiplied. Samples with 0–2 points were classified into the low expression group, while those with ≥3 points were classified into the high expression group.

### 2.4 Statistical Analysis

The patients’ clinical characteristics were summarized using descriptive statistics. The UCD and MCD were compared using the *X* ^2^ test, Fisher’s exact test, and t-test. The Kaplan-Meier method was used to analyze the survival curves, and the differences were compared using the log-rank test. All statistical analyses were performed using the SPSS software (Version 21.0). A *P*-value of <0.05 was considered significant.

## 3 Results

### 3.1 Clinical Characteristics and Manifestations

A total of 19 patients had UCD, with an average age of 39 years, while 41 had MCD, with an average age of 36 years. Among the MCD patients, two had TAFRO syndrome. The most common pathology type of UCD was hyaline vascular, whereas the plasma cell type and mixed type were highly expressed in MCD (*P*<0.001). No significant difference was observed between the MCD and UCD groups in terms of age and sex. The most common initial symptom of UCD was lymphadenopathy, especially in the neck, which was also observed in the axillary, groin, breast, and other parts, and UCD was more prevalent than MCD (*P*=0.019). Fever (38°C) was the initial symptom of MCD, which was accompanied by night sweats, fatigue, and so on (*P*=0.034). Some patients remained asymptomatic regardless of the CD type, but UCD was more common than MCD (*P*=0.031) (**Table 1**).

**Table 1:** Clinical characteristics and manifestations of patients with UCD and MCD.

	UCD (n = 19)	MCD (n = 41)	*P* Value
Age(years)	39 ± 15	36 ± 15	0.453
Gender			0.781
Female	9	21	
Male	10	20	
Histopathological subtype			**<0.001**
Plasmacytic variant	0	17	
Hyaline vascular variant	19	13	
Mixed cellular variant	0	11	
No symptoms	4	1	**0.031**
Lymphadenopathy	11	12	**0.019**
Neck	8	6	
Axilla	1	2	
Groin	1	0	
Breast	1	1	
Submandibular	0	2	
Posterior auricular	0	1	
Fever	2	17	**0.034**
Fatigue	0	3	0.546
Edema	1	4	1.000
Mouth ulcer	0	2	1.000
Abdominal pain	1	2	1.000
Cough, chest tightness	0	1	1.000
Foam urine	0	1	1.000

Bold values is statistically meaningful (P < 0.05).

### 3.2 Laboratory Tests and Virus Detection

By analyzing the detailed laboratory test information of patients with CD, we found that MCD patients were more likely to have anemia, hypoproteinemia, abnormal albumin/globulin (A/G) ratio, abnormal platelet count, and elevated lactate dehydrogenase (LDH) and urinary protein levels than UCD patients (*P*<0.05) (**Table 2**). Twenty-seven patients underwent a series of tests for common types of viruses, including Coxsackie virus, measles virus, cytomegalovirus, and herpes simplex virus; meanwhile, 55 patients underwent a series of tests for common infectious diseases. All patients tested negative for HIV serum antibodies. EBV was found in 87.0% of the tested patients with MCD and in 75.0% of patients with UCD (*P*=0.025). Similar proportions of patients tested positive for cytomegalovirus (**Table 3**).

**Table 2:** Laboratory test information of patients with UCD and MCD.

	UCD (n = 19)	MCD (n = 41)	*P* Value
Anemia	3	22	**0.010**
Abnormal WBC count	3	7	1.000
Abnormal neutrocyte count	1	8	0.249
Abnormal lymphocyte count	4	11	0.755
Abnormal platelet count	4	23	**0.013**
Hypoalbuminemia	1	18	**<0.001**
Elevated globulin	1	4	1.000
Abnormal albumin/globulin(A/G)	6	27	**0.024**
Elevated LDH	0	7	**<0.001**
Elevated β2-MG	1	10	0.086
Elevated alkaline phosphatase	1	6	0.414
Elevated uric acid	3	9	0.735
Elevated creatinine	1	3	1.000
Elevated urinary protein	1	12	**0.045**

Bold values is statistically meaningful (P < 0.05).

**Table 3:** Virological results in UCD and MCD patient.

Virology	UCD (n=19)		MCD (n=41)		*P* value
	Tested in n patients	Positive (%)	Tested in n patients	Positive (%)	
HIV	18	0	37	0	1.000
HBV	18	0	37	3	0.543
HCV	18	0	37	1	1.000
EBV	4	3	23	20	**0.025**
CoxsackieV	4	0	23	2	1.000
MeaselesV	4	0	23	3	1.000
Cytomegalovirus	4	3	23	20	**0.025**
Herpes SimplexV	4	1	23	0	0.148

Bold values is statistically meaningful (P < 0.05).

### 3.3 Imaging Examination

**Figure 2:**
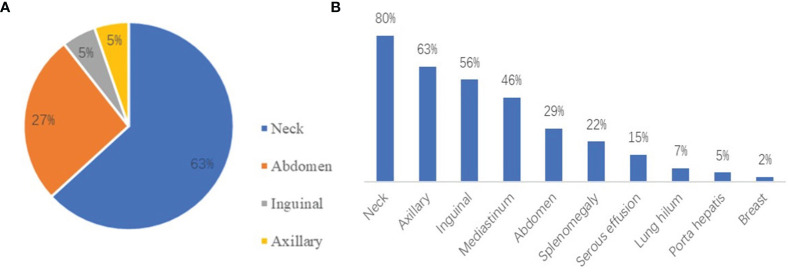
**(A)** The distribution of lymphadenopathy among patients with HIV-negative UCD. **(B)** The locations of coexistent lymphadenopathies and other signs among patients with MCD.

According to the computed tomography (CT) and ultrasound images of patients, the most common region of lymphadenopathy was the neck in both UCD patients (65.0%) and MCD patients (80.5%). In addition, splenomegaly (22.0%) and serous effusion (14.6%) were observed in patients with MCD (**Figure 2**). Positron emission tomography-CT (PET-CT) was performed in 17 patients. Comparing the maximum standard uptake value (SUVmax) according to multicentricity, SUVmax was significantly higher in the MCD group than in the UCD group (6.8 ± 4.2 and 2.8 ± 0.7, respectively; *P*<0.001).

### 3.4 Immunohistochemistry

#### 3.4.1 Conventional IHC

CD3, CD20, CD21, and Ki-67 were detected in most CD patients. CD3, CD20, and CD21 were detected in all (100%) patients with UCD and in 90% of patients with MCD. Ki-67 was expressed in all patients, and the mean positive rate was approximately 23%. A significant difference was observed in the expression of Bcl-2 between UCD patients and MCD patients (*P*=0.034, **Supplemental Table**).

#### 3.4.2 Expression of mTOR Pathway-Related Proteins

**Figure 3:**
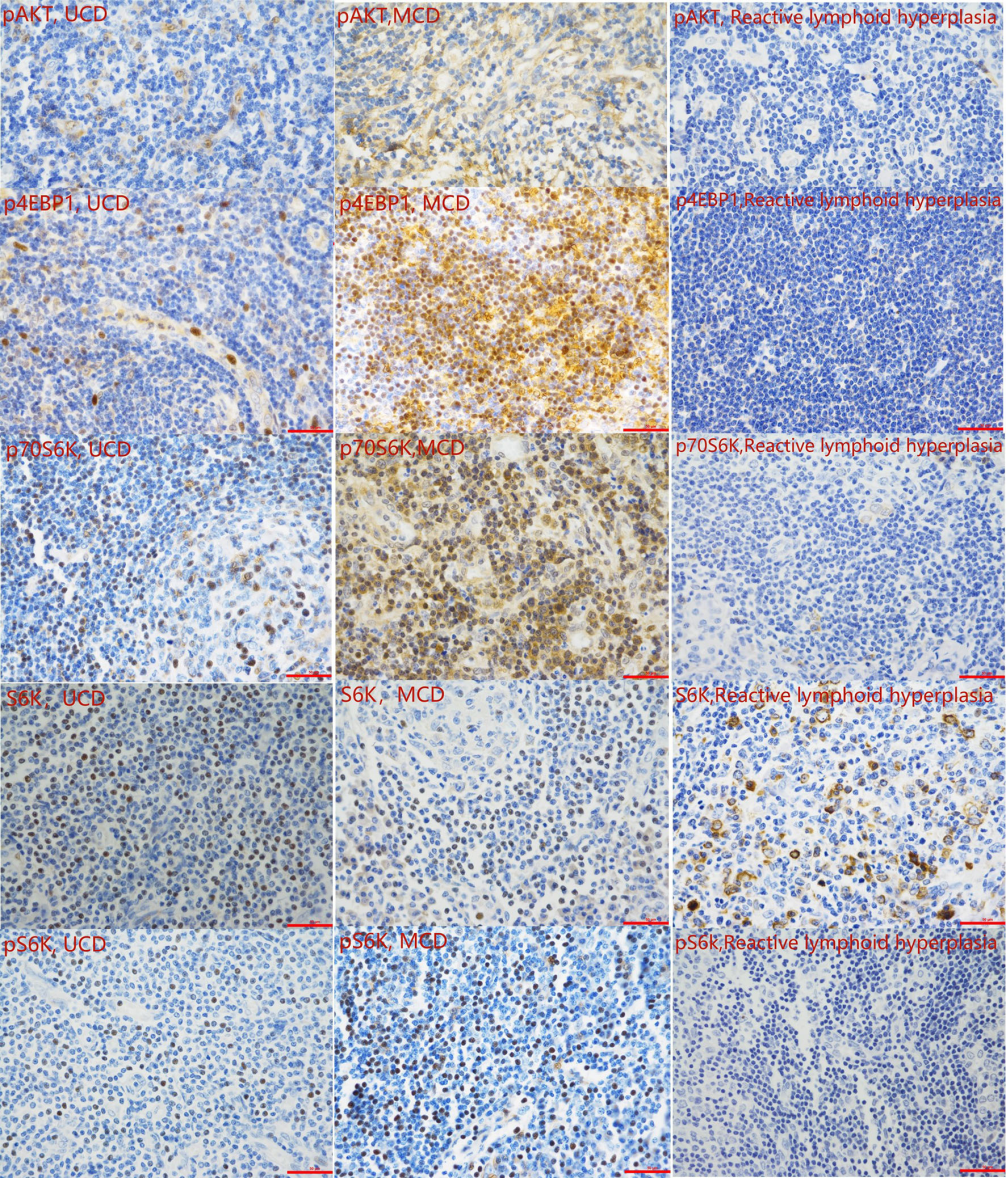
Representative immunohistochemistry images of the mTOR pathway activity analysis (400 magnification).

To verify the activity of the mTOR pathway, the phosphorylation levels of AKT, 4EBP1, p70S6K, and S6K, and levels of unphosphorylated S6k were measured by immunohistochemistry in the tissue samples of 31 patients with CD and 10 patients with reactive lymphoid hyperplasia (**Figure 1B**). IHC results showed that the expression levels of pAKT, p4EBP1, p70S6K, and pS6k in the tissue samples of CD patients was significantly higher than those in patients with reactive lymphoid hyperplasia (*P*=0.001, 0.007, 0.039, and 0.007, respectively) (**Table 4A**, **Figure 3**). We also analyzed the difference in the expression levels of mTOR pathway-related proteins between UCD patients and MCD patients. Expression levels of p4EBP1, p70S6K, and pS6k were higher in MCD patients than in UCD patients (*P*=0.029, <0.001, and <0.001, respectively) (**Table 4B**). Inconsistency was detected in the difference in pAKT expression. Further analysis showed that the expression levels of pAKT in UCD and MCD patients were higher than those in patients with reactive lymphoid hyperplasia (*P*=0.011,0.002 respectively).

**Table 4A:** Expression differences between Castleman and Reactive lymphoid hyperplasia.

		CD (n=31)	Reactive lypmhoid hyperplasia (n=10)	*P* value
pAKT	High	9	1	**0.001**
	Low	22	9	
P4EBP1	High	16	0	**0.007**
	Low	15	10	
P70S6k	High	20	1	**0.039**
	Low	11	9	
S6k	High	13	3	0.712
	Low	18	7	
pS6K	High	16	0	**0.007**
	Low	15	10	

Bold values is statistically meaningful (P < 0.05).

**Table 4B:** Expression differences between UCD and MCD.

		UCD (n=12)	MCD (n=19)	*P* value
pAKT	High	4	5	0.704
	Low	8	14	
P4EBP1	High	3	13	**0.029**
	Low	9	6	
P70S6k	High	3	17	**<0.001**
	Low	9	2	
S6k	High	3	10	0.158
	Low	9	9	
pS6K	High	1	15	**<0.001**
	Low	11	4	

Bold values is statistically meaningful (P < 0.05).

### 3.5 Univariate and Survival Analyses of CD

**Figure 4:**
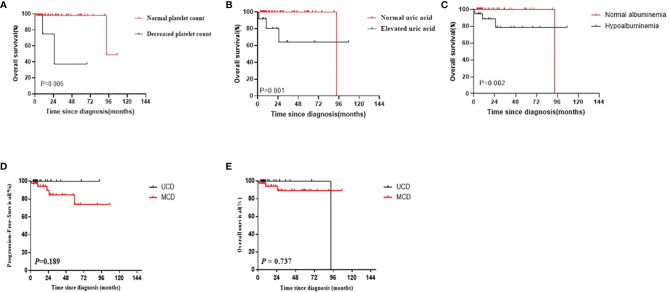
**(A–C)** The survival curves of different risk factors among patients with CD **(A)** Platelet count; **(B)** Uric acid; **(C)** Hypoalbuminemia). **(D, E)** PFS and OS curves of patients with CD by multicentricity (**D**: PFS, **E**: OS).

Using the Kaplan–Meier method, three risk factors were identified among patients with CD: thrombocytopenia, elevated uric acid levels, and hypoalbuminemia (*P*=0.006, 0.001, and 0.002, respectively; **Figures 4A–C**). No significant differences were observed in other investigated factors including LDH, β2-MG, clinical complaint, B symptoms, hepatosplenomegaly, serous cavity effusion, anemia, and A/G ratio. Moreover, no significant differences were observed in the PFS and OS survival curves between UCD patients and MCD patients (*P*=0.189 and *P*=0.737, respectively; **Figures 4D, E**).

## 4 Discussion

CD is an uncommon lymphoproliferative disorder with remarkably heterogeneous clinicopathologic findings and is included in *First National List of Rare Diseases* issued by the Chinese government (10). Because of the low prevalence, the clinical studies conducted to investigate CD were primarily case reports and small series trials, posing challenges for the diagnosis and management of patients with CD (11). Using the detailed clinical, laboratory, imaging, and virology data of 60 patients with CD, we were able to provide a comprehensive evaluation of CD to improve the clinicians’ understanding of this condition. Furthermore, we presented a comprehensive IHC analysis of the mTOR pathway activity in patients with CD to better understand its underlying mechanism and to develop effective therapeutic targets in order to improve patients’ survival.

UCD typically involves single lymph nodes or single regional lymph nodes. Patients with UCD are usually asymptomatic; however, the enlargement of lymph nodes can trigger the occurrence of corresponding symptoms (12). MCD involves the enlargement of multiple regional lymph nodes and is often accompanied by inflammatory symptoms, such as fever, night sweats, fatigue, weight loss, and dropsy of the serous cavity (13). The laboratory test results of UCD patients are usually normal, but anemia, increased C-reactive protein (CRP) levels, increased erythrocyte sedimentation rate (ESR), and other abnormalities can be present (14). MCD is often accompanied by a systemic inflammatory response, which is characterized by a variety of laboratory and imaging abnormalities, such as anemia, hypoalbuminemia, elevated CRP and ESR levels, abnormal liver and kidney function, and serous effusion (15). In our study, anemia, abnormal platelet count, abnormal A/G, and elevated LDH levels were significantly more common in MCD patients than in UCD patients (all *P*<0.05). EBV and cytomegalovirus were more commonly detected in MCD patients than in UCD patients based on the results virological tests; hence, we cannot confirm the causal relationship between virus infection and the extent of disease. Chih-Hao Chen et al. published their experience of 20 cases of Castleman disease, which indicated that more EBV-positive cells in germinal centers are associated with increased vascularity and smaller tumor size. Anti-vessel growth therapy plays a potential role to control the disease (16). The localization and distribution of UCD and MCD are shown in **Figure 2**. The neck was the most common site of lymphadenopathy in patients with UCD (63%) and those with MCD (80%). PET-CT can be a useful diagnostic imaging modality for assessing the involved lymph nodes in patients with CD. CD demonstrates an increased but variable range of 18F-FDG uptake (17). In several case reports, CD showed as SUV_max_ of over 2.0. In a subgroup of 17 patients, PET-CT scan was performed, and the SUV_max_ was significantly higher in the MCD group than in the UCD group (18). According to multicentricity, SUV_max_ was significantly higher in the MCD group than in the UCD group in our study (6.8 ± 4.2, and 2.8 ± 0.7, respectively; *P*<0.001). Bcl-2 is a key protein that regulates apoptosis. It can promote the growth of tumor cells by cooperating with the ras proto-oncogene (19). Bcl-2 overexpression is observed in various human tumor tissues and cells. The positive rate of Bcl-2 in MCD patients was higher than that in UCD patients (*P*=0.040), which were considered correlated with the high proliferative activity of MCD.

Most CD patients have a good prognosis (20). Furthermore, the survival of patients with UCD was better than that of patients with MCD, but the difference was not significant, which may be due to the small sample size or presence of statistical bias. However, the condition of some patients with CD progresses rapidly, and their prognosis becomes relatively poor (21, 22). Hence, it is crucial to identify the prognostic factors of different classifications to improve treatment decisions and determine the prognosis early (11). Thrombocytopenia, increased uric acid levels, and hypoalbuminemia were identified as poor prognostic factors of CD.

Megakaryocytes usually produce more platelets when IL-6 is boosted, which contradicts the occurrence of thrombocytopenia (23). TAFRO syndrome, which is characterized by thrombocytopenia, anasarca, fever, reticulin fibrosis, and organomegaly, is a subtype with the worst prognosis (24, 25). Thrombocytopenia occurred in four patients, while only two patients were diagnosed with TAFRO syndrome. The other two patients had no other relevant anomalies except thrombocytopenia. Therefore, we confirmed that thrombocytopenia is a poor diagnostic factor for CD, not just putting down to TAFRO syndrome. However, the reason for this remains unclear. The increase in uric acid level is related to worsening of renal function, which has been identified as a possible risk factor in previous large-scale studies (26, 27). Hypoalbuminemia was also proven to be a poor diagnosis factor as it was associated with other conditions, including renal function deterioration, splenomegaly, effusion, and so on, which was brought forth in a previous study (11).

Surgical resection is the first line of treatment for UCD, and the cure rate of surgical resection is approximately 90% (28). There is no standard treatment for relapse or refractory UCD patients. As for iMCD patients, IL-6 blockers can decrease symptoms and lymphadenopathy (29, 30). Approximate 66% of the patients in a randomized controlled trial did not respond to the therapy, and mTOR has been recommended as a second-line treatment for iMCD patients (31). This is a follow-up study to previous work in the CD field related to mTOR pathway. For further analysis of the mTOR pathway in CD, we analyzed the related proteins, including pAKT, p4EBP1, p70S6K, S6K, and pS6k. The results indicated that the activity of the mTOR pathway was higher than that of reactive lymphoid hyperplasia, and some UCD specimens were tested positive. We suspected that the increasing IL-6 may activate the mTOR pathway causing CD to a certain extent. The mTOR inhibitors may be effective in all types of CD, both UCD and MCD.

Because of the low prevalence, the types of clinical studies that have investigated CD are primarily case reports and small series trials, posing challenges for the diagnosis and management of patients with CD. There is no established standardized treatment protocol, and clinicians lack sufficient knowledge to manage patients rationally. The present study increases our understanding of CD and contributes to more detailed clinical information on this rare disease. We initially put forward that thrombocytopenia is a poor diagnostic factor for CD, which serves as a basis when making appropriate clinical decisions. We also explored the mTOR pathway activity in CD patients, offering some elucidation for the management and treatment of CD patients. Larger studies are required to clarify the molecular abnormalities and potential therapeutic targets of this uncommon condition.

## Conclusion

Thrombocytopenia was the independent poor prognostic factor for CD was first proposed, as well as elevated uric acid levels, hypoalbuminemia. And the activity of the mTOR pathway was higher than reactive lymphoid hyperplasia which served as control group, and some UCD specimens were tested positive.

## Data Availability Statement

The raw data supporting the conclusions of this article will be made available by the authors, without undue reservation.

## Author Contributions

SQ, MJD, and XZ designed the research. All investigators and their respective research teams recruited and followed up the patient. HH, YZ, MD, MJD, and LZ collected and analyzed research data. SQ and ZW wrote and edited the manuscript. GW and WL are pathologists who interprets the immunohistochemical results. All authors were involved at each stage of manuscript preparation and approved the final version. All authors contributed to the article and approved the submitted version.

## Funding

The authors would like to thank the financial support provided by National Natural Science Foundation of China (82070210) and Major Medicine Scientific and Technology Project of Henan Province (NO.SBG 202001008).

## Conflict of Interest

The authors declare that the research was conducted in the absence of any commercial or financial relationships that could be construed as a potential conflict of interest.

## Publisher’s Note

All claims expressed in this article are solely those of the authors and do not necessarily represent those of their affiliated organizations, or those of the publisher, the editors and the reviewers. Any product that may be evaluated in this article, or claim that may be made by its manufacturer, is not guaranteed or endorsed by the publisher.

## Supplementary Material

Supplementary Table 1 Conventional IHC difference between UCD and MCD.
