# Supplementary material for: Clinical and Molecular Characteristics of 60 Patients With Human Immunodeficiency Virus-Negative Castleman Disease
Source: Front Immunol. 2022 May 17;13:899073. doi: 10.3389/fimmu.2022.899073 (PMC9152317; doi:10.3389/fimmu.2022.899073)
Supplement: Supplementary Table 1 — Conventional IHC difference between UCD and MCD. [file Table_1.docx]

**Supplement Table**

Table 5 Conventional IHC difference between UCD and MCD

| Immunohistochemical index | UCD（n=19） | | MCD（n=41） | | P value |
| --- | --- | --- | --- | --- | --- |
|  | Tested in n patients | Positive (%) | Tested in n patients | Positive (%) |  |
| CD3 | 18 | 18（100.0%） | 36 | 33（91.7%） | 0.543 |
| CD5 | 10 | 9（90.0%） | 11 | 10（90.9%） | 1.000 |
| CD10 | 14 | 12（85.7%） | 17 | 11（64.7%） | 0.240 |
| CD20 | 18 | 18（100.0%） | 36 | 32（88.9%） | 0.289 |
| CD21 | 17 | 17（100.0%） | 35 | 35（100.0%） | 1.000 |
| CD30 | 8 | 4（50%） | 10 | 4（40.0%） | 0.520 |
| CD34 | 5 | 5（100.0%） | 2 | 2（100.0%） | 1.000 |
| CD38 | 9 | 8（88.9%） | 22 | 22（100.0%） | 0.290 |
| CD43 | 7 | 7（100.0%） | 6 | 5（83.4%） | 0.462 |
| CD68 | 3 | 2（66.7%） | 2 | 2（100.0%） | 1.000 |
| CD79a | 7 | 7（100.0%） | 15 | 15（100.0%） | 1.000 |
| CD123 | 4 | 4（100.0%） | 6 | 5（83.3%） | 1.000 |
| CD138 | 2 | 2（100.0%） | 18 | 17（94.4%） | 1.000 |
| Kappa | 3 | 2（66.7%） | 21 | 21（100.0%） | 0.125 |
| Lambda | 3 | 2（66.7%） | 22 | 21（95.5%） | 0.230 |
| Ki-67 | 17 | 17（100.0%） | 36 | 36（100.0%） | 1.000 |
| Bcl-2 | 11 | 11（83.3%） | 23 | 15（65.2%） | **0.034** |
| Bcl-6 | 10 | 9（90.0%） | 8 | 7（87.5%） | 1.000 |
| MUM-1 | 1 | 1（100.0%） | 8 | 7（87.5%） | 1.000 |
| IgG | 0 | 0 | 11 | 11（100.0%） | 1.000 |
| IgG4 | 0 | 0 | 11 | 10（90.9%） | 1.000 |
| CyclinD1 | 10 | 1（10.0%） | 9 | 1（11.1%） | 1.000 |
